# SGK1 Inhibits Autophagy in Murine Muscle Tissue

**DOI:** 10.1155/2018/4043726

**Published:** 2018-04-22

**Authors:** Theresia Zuleger, Julia Heinzelbecker, Zsuzsanna Takacs, Catherine Hunter, Jakob Voelkl, Florian Lang, Tassula Proikas-Cezanne

**Affiliations:** ^1^Department of Molecular Biology, Interfaculty Institute of Cell Biology, Eberhard Karls University Tuebingen, Tuebingen, Germany; ^2^International Max Planck Research School “From Molecules to Organisms”, Tuebingen, Germany; ^3^Institute of Physiology, Physiology I, Eberhard Karls University Tuebingen, Tuebingen, Germany

## Abstract

**Background/Aims:**

As autophagy is linked to several pathological conditions, like cancer and neurodegenerative diseases, it is crucial to understand its regulatory signaling network. In this study, we investigated the role of the serum- and glucocorticoid-induced protein kinase 1 (SGK1) in the control of autophagy.

**Methods:**

To measure autophagic activity *in vivo*, we quantified the abundance of the autophagy conjugates LC3-PE (phosphatidylethanolamine) and ATG12-ATG5 in tissue extracts of SGK1 wild-type (*Sgk1* ^+/+^) and knockout (*Sgk1* ^−/−^) mice that were either fed or starved for 24 h prior sacrifice. *In vitro,* we targeted SGK1 by RNAi using GFP-WIPI1 expressing U-2 OS cells to quantify the numbers of cells displaying newly formed autophagosomes. In parallel, these cells were also assessed with regard to LC3 and ULK1 by quantitative Western blotting.

**Results:**

The abundance of both LC3-PE (LC3-II) and ATG12-ATG5 was significantly increased in red muscle tissues of SGK1 knockout mice. This was found in particular in fed conditions, suggesting that SGK1 may keep basal autophagy under control in red muscle *in vivo*. Under starved conditions, significant differences were observed in SGK1-deficient white muscle tissue and, under fed conditions, also in the liver. *In vitro*, we found that SGK1 silencing provoked a significant increase of cells displaying WIPI1-positive autophagosomes and autophagosomal LC3 (LC3-II). Moreover, autophagic flux assessments revealed that autophagic degradation significantly increased in the absence of SGK1, strongly suggesting that SGK1 inhibits both autophagosome formation and autophagic degradation *in vitro*. In addition, more ULK1 protein lacking the inhibitory, TORC1-specific phosphorylation at serine 758 was detected in the absence of SGK1.

**Conclusions:**

Combined, our data strongly support the idea that SGK1 inhibits the process of autophagy. Mechanistically, our data suggest that SGK1 should act upstream of ULK1 in regulating autophagy, and we hypothesize that SGK1 contributes to the regulation of ULK1 gene expression.

## 1. Introduction

Macroautophagy (referred to as autophagy) is a catabolic pathway that degrades cytosolic components, like proteins and damaged organelles. The cargo is engulfed by newly formed double-membrane vesicles, termed autophagosomes, which fuse with lysosomes for cargo degradation. The degraded monomers are released to the cytoplasm for recycling processes engaging anabolic pathways. Dysregulation of autophagy often results in pathological conditions like cancer and metabolic and neurodegenerative diseases [1, 2].

Autophagosome formation is tightly regulated by ATG (autophagy-related) proteins [2]. Amongst the ATG proteins, the ULK1 protein is crucial for the initiation of autophagosome formation and interacts with both the AMP-activated protein kinase (AMPK) and the mechanistic target of rapamycin complex 1 (mTORC1), the energy and nutrient sensors of the eukaryotic cell. Under low energy levels, AMPK activates, and under nutrient-rich conditions, mTORC1 inhibits ULK1 and autophagy [3, 4]. The generation of phosphatidylinositol 3-phosphate (PI3P) by the phosphatidylinositol 3-kinase class III (PI3KC3) is essential for the nucleation of autophagosome formation and occurs downstream of ULK1 [5, 6]. PI3P recruits the WD-repeat protein interacting with phosphoinositide (WIPI) proteins (WIPI1–4) to the membrane origin for autophagosome formation [7]. Upon autophagy induction, the WIPI proteins are recruited to autophagosomal membranes; visualizing their localization has been established to assess autophagic activity by fluorescence microscopy (WIPI puncta formation) [8, 9]. Conjugation of LC3 to phosphatidylethanolamine (PE) by the ATG12 and LC3 ubiquitin-like conjugation systems is necessary for the elongation and closure of the autophagosomal membrane [2]. Lipidation can be measured by distinguishing the nonlipidated, cytosolic form of LC3 (LC3-I) from the lipidated, membrane-bound form of LC3 (LC3-PE or LC-II) by Western blotting. Increased levels of the ATG12-ATG5 conjugate and lipidated LC3 suggest increased autophagy [10, 11].

The serine/threonine-specific kinase SGK1 [12] was identified as a serum- and glucocorticoid-responsive gene in rat mammary epithelial tumour cells [13] and as a cell volume-sensitive transcript in a human hepatocellular carcinoma cell line [14]. SGK1 expression and activity can be induced via various stimuli like hormones, cytokines, and cellular stress [15]. SGK1 is regulated by the PI3K pathway, serine 422 is phosphorylated by mTORC2 [16, 17], which facilitates the phosphorylation of threonine 265 by PDK1 [15, 18]. Among others, SGK1 is involved in osmoregulation, transcription factor regulation, and cell proliferation [15, 19, 20]. Despite its broad function, SGK1-deficient mice develop a mild phenotype, which only becomes apparent under a challenging condition [21].

As both mTORC1 [22] and mTORC2 [16, 17] activate SGK1 and inhibit autophagy, SGK1 is considered an inhibitor of autophagy. H_2_S was shown to suppress autophagy via stimulation of SGK1 [23], and inhibition of SGK1 induces LC3 lipidation and BECN1 (member of the PI3KC3 complex) expression in human glioblastoma cells [24]. SGK1 also inhibits FOXO3A [25–27], a transcription factor of autophagy genes [28]. In spite of the previous data, the mechanism of autophagy regulation by SGK1 is not understood. Here we show that red muscle tissue in SGK1 knockout mice has increased LC3-II and ATG12-ATG5 levels, suggesting increased autophagic activity. *In vitro*, we observed that SGK1 silencing provoked a significant increase of the autophagic flux as analyzed by GFP-WIPI1 puncta formation and LC3 lipidation. In addition, we found that the abundance of ULK1 protein lacking the inhibitory TORC1-dependent phosphorylation site increased in the absence of SGK1. Based on our findings, we entertain the hypothesis that SGK1 may control autophagy through ULK1.

## 2. Methods

### 2.1. *Sgk*−/− Mice

All animal experiments were approved by local authorities, adhered to the German law for the welfare of animals, and conducted in the Florian Lang laboratory. Mice deficient in SGK1 (*Sgk1*−/−) were bred and genotyped as previously described [21]. The experiments were conducted in mice on an original SV129 background [21]. SGK1 knockout (*Sgk1*−/−) and SV129 wild-type control mice (*Sgk1*+/+) of identical age (3 months) were fed *ad libitum* or starved for 24 h by keeping free access to tap drinking water. For subsequent tissue dissections, mice were anaesthetized with isoflurane and sacrificed by cervical dislocation. Organs were immediately harvested and flash frozen in liquid nitrogen.

### 2.2. Protein Extracts and Western Blotting Using Mouse Tissues

Tissue samples from the liver, kidney, heart, white muscle, red muscle, and aorta were collected from SGK1 knockout mice (*Sgk1*−/−, *n* = 6; 2 females, 4 males) and wild-type control (*Sgk1*+/+, *n* = 6; 2 females, 4 males) mice [21] that were either fed (*n* = 3) or starved (*n* = 3). Depending on the weight, samples were mixed with 1–1.5 ml RIPA buffer (150 mM NaCl, 1 mM EDTA, 10 mM Tris pH 8.0, 0.1% SDS, 1% deoxycholic acid, 1% NP-40) supplemented with protease inhibitor (Roche, 04693159001) and sonicated four times at 30.000/min for 15 seconds with a PT-DA 2105/2EC rotor (Polytron) in order to extract the proteins. After sonication, the samples were centrifuged at 14.000 rpm for 20 minutes at 4°C. 500 *μ*l of the supernatant was mixed with 500 *μ*l 2x Laemmli buffer (50 mM Tris pH 6.8, 1.25 mM EDTA pH 8.0, 12.5% glycerine, 2% SDS, 50 mM DTT, 2.5% *β*-mercaptoethanol, 0.025% bromophenol blue) and boiled for 5 minutes. The proteins were separated by SDS-PAGE and blotted on PVDF membranes (Millipore, IPVH00010). For LC3 detection 15% and for ATG12 detection 10%, SDS-PAGE gels were prepared. The following antibodies were used: LC3 (NanoTools, 0231-100/LC3-5F10), ATG12 (Abgent, ASC10625), *α*-tubulin (Sigma-Aldrich, T6074), anti-mouse IgG-HRP (Cell Signaling, 7076), and anti-rabbit IgG-HRP (Cell Signaling, 7074). LC3-positive control was purchased from NanoTools. ECL detection was performed with Immobilon Western Chemiluminescent HRP Substrate (Millipore, WBKLS0100). Images were taken using the Fusion SL Vilber Lourmat device. The FUSION-CAPT Advance Software (Vilber Lourmat) was used for quantification.

### 2.3. Cell Culture

U-2 OS cells stably expressing GFP-WIPI1 were cultured in DMEM (Life Technologies, 31966) supplemented with 10% FCS (PAA, A15–101), 100 U/ml penicillin/100 *μ*g/ml streptomycin (Life Technologies, 15140), 0.6 mg/ml G418 (Life Technologies, 11811098) at 37°C, and 5% CO_2_.

### 2.4. Transient Transfections

Transient knockdown experiments with siRNA (SGK1 siRNA (h), Santa Cruz, sc-38913; control siRNA-A, Santa Cruz, sc-37007) were conducted by using Lipofectamine RNAiMAX (Invitrogen; 13778-075) according to the manufacturer's reverse transfection protocol. Briefly, in each well of a 24-well plate, 50 nM siRNA and 1 *μ*l Lipofectamine RNAiMAX were diluted in 96 *μ*l OPTI.MEM (Life Technologies; 51985-026) and the transfection solution incubated for 20 min at room temperature. Finally, 40,000 (for WIPI1 puncta formation analysis) or 50,000 (for Western blotting) cells (U-2 OS GFP-WIPI1) in 500 *μ*l DMEM/10% FCS were added to the transfection solution for 63 h. Knockdown events were verified by immunoblotting.

### 2.5. WIPI1 Puncta Formation Analysis

After transient transfection, 3 h treatments were performed under fed (DMEM/10% FCS) or serum and amino acid starvation (EBSS, Earl's balanced salt solution, Sigma-Aldrich, E2888) conditions in the presence and absence of 200 nM bafilomycin A1 (AppliChem, A7823). The cells were fixed with 3.7% PFA and mounted on slides using ProLong (Life Technologies, P36930). Up to 1541 cells (per treatment) from 3 independent experiments were counted manually by fluorescence microscopy (Zeiss, Axiovert 200M), and the percentages of cells positive for GFP-WIPI1 puncta were calculated. Representative images were taken with an Axiocam MRm camera using a 40x objective.

### 2.6. Protein Extracts and Western Blotting Using U-2 OS Cells

GFP-WIPI1 expressing U-2 OS cells were treated as described above for WIPI1 puncta formation analysis, except that after the 3 h treatment period, cells were lysed with hot 2x Laemmli buffer (50 mM Tris pH 6.8, 1.25 mM EDTA pH 8.0, 12.5% glycerine, 2% SDS, 50 mM DTT, 2.5% *β*-mercaptoethanol, 0.025% bromophenol blue), scraped into Eppendorf tubes, and sheered with a 23G injection needle. 40 *μ*l of the extracts was loaded on a 10% or 15% SDS-PAGE gel, blotted on to a PVDF membrane (Millipore, IPVH00010), and incubated with the following antibodies: SGK1 (Cell Signaling, 12103), LC3 (NanoTools, 0231–100/LC3-5F10), phospho-ULK1 (S757) (Cell Signaling, 6888), ULK1 (Cell Signaling, 8054), *α*-tubulin (Sigma-Alderich, T6074), anti-mouse IgG-HRP (Cell signaling, 7076), and anti-rabbit IgG-HRP (Cell Signaling, 7074). Subsequently, ECL detection was performed with SuperSignal West Femto Maximum Sensitivity Substrate (Thermo Scientific, 34096). Images were taken using the Fusion SL Vilber Lourmat device.

### 2.7. Statistics

Western blot and fluorescence microscopy results were analysed using SAS JMP 11.1® and two-tailed heteroscedastic *t*-testing. Statistical analyses with *p* values can be found in the supplementary data set (Suppl. Data Set (available here)).

## 3. Results

### 3.1. Autophagy Is Increased in SGK1-Deficient Mice

**Figure 1:**
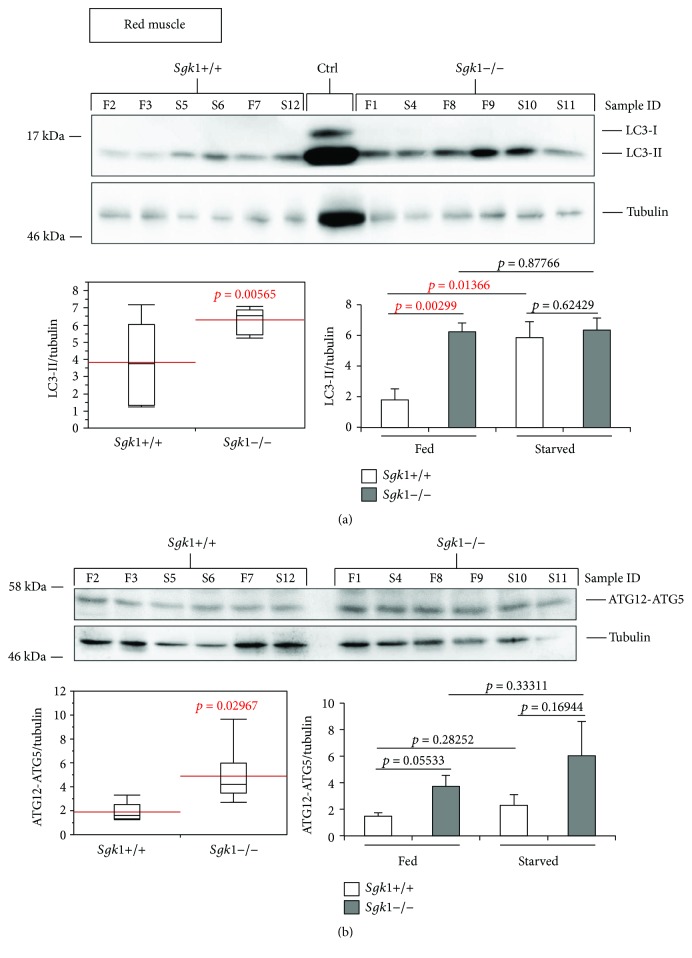
LC3-II and ATG12-ATG5 abundances are significantly increased in the red muscle tissue of Sgk1 knockout mice. LC3 lipidation was analysed by Western blotting (upper panel) and quantified (lower panels) in protein extracts from the red muscle of Sgk1 knockout (*Sgk1*−/−) and wild-type (*Sgk1*+/+) mice (*n* = 6) (a). Of those, mice were either fed (F, *n* = 3) or starved (S, *n* = 3) as indicated (sample ID). LC3-positive control (Ctrl) was used to indicate the migration of nonlipidated LC3-I and lipidated LC3-II. Red lines on the box plots (lower panel, left) represent the mean values of all 6 Sgk1 knockout (*Sgk1*−/−) and wild-type (*Sgk1*+/+) mice (lower panel, left) or with regard to fed and starved conditions (lower panel, right). *p* values are provided. In parallel, the abundance of the ATG12–ATG5 (ATG12–5) conjugate was likewise assessed (b). Supporting data is provided (Suppl. Data Set).

**Figure 2 fig2:**
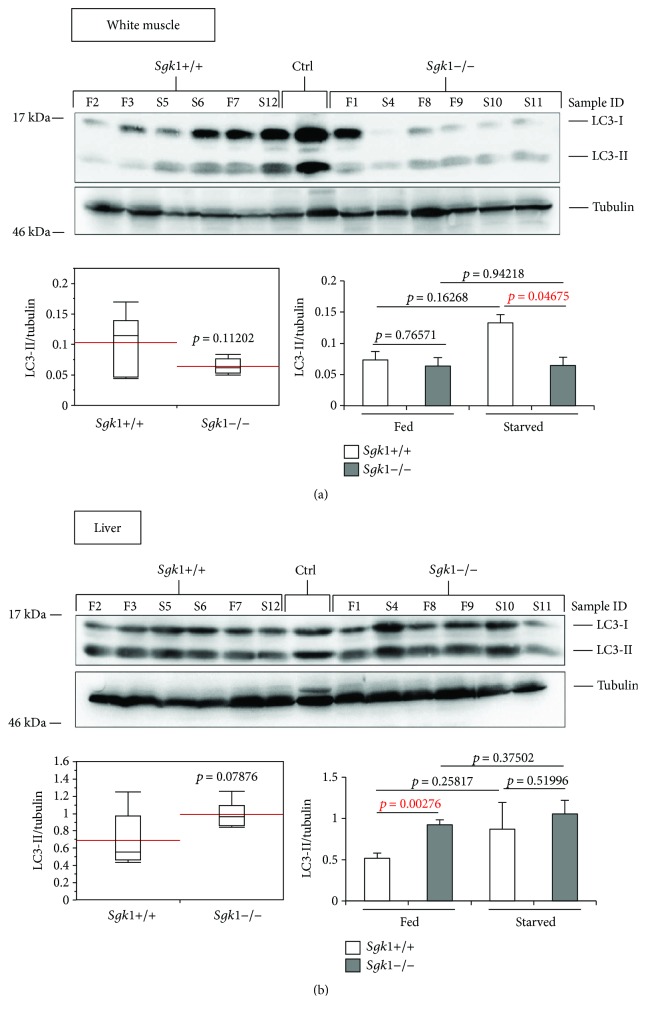
LC3-II abundance appears weakly altered in the white muscle and liver tissue of Sgk1 knockout mice. LC3 lipidation was analysed by Western blotting (upper panel) and quantified (lower panels) in protein extracts from the white muscle (a) or liver (b) of Sgk1 knockout (*Sgk1*−/−) and wild-type (*Sgk1*+/+) mice (*n* = 6). Of those, 3 mice were either fed (F) or starved (S) as indicated (sample ID). LC3-positive control (Ctrl) was used to indicate the migration of nonlipidated LC3-I and lipidated LC3-II. Red lines on the box plots (lower panel, left) represent the mean values of all 6 Sgk1 knockout (*Sgk1*−/−) and wild-type (*Sgk1*+/+) mice (lower panel, left) or with regard to fed and starved conditions (lower panel, right). *p* values are provided. Supporting data is provided (Suppl. Data Set).

**Figure 3 fig3:**
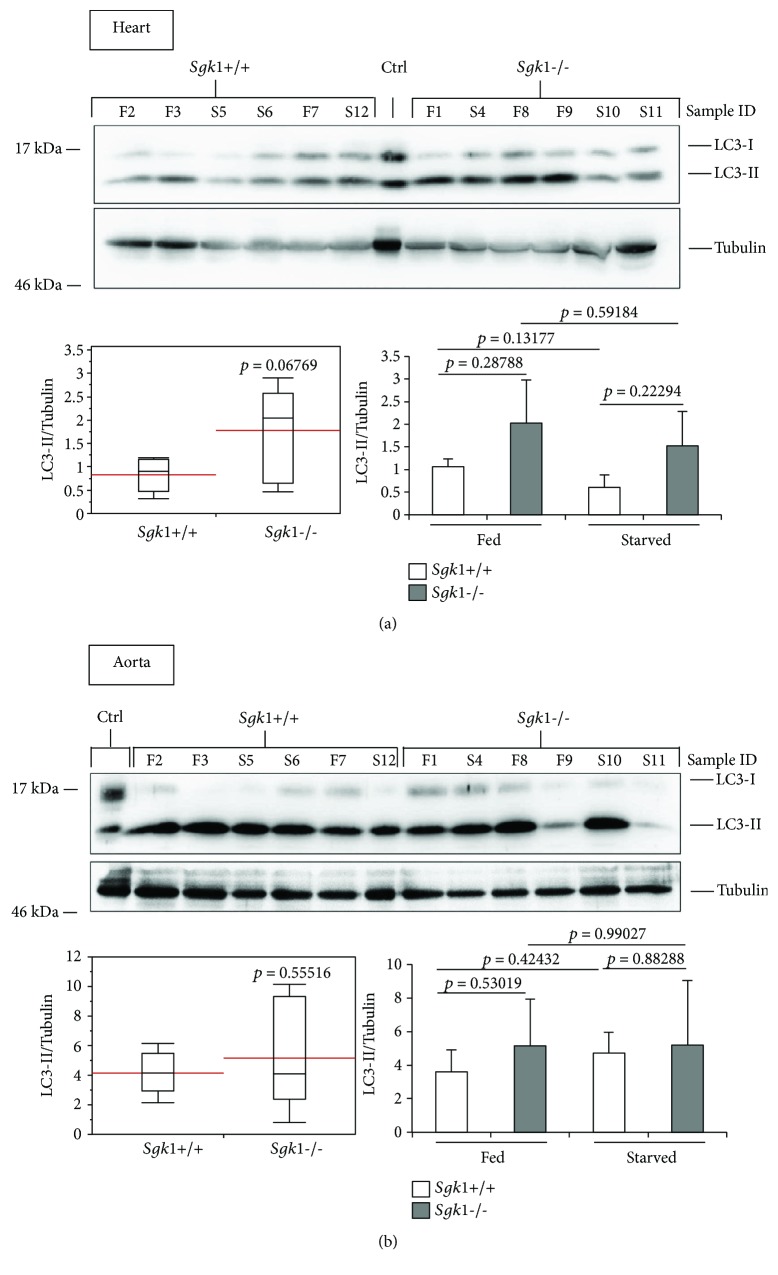
LC3 lipidation is unaltered in the heart and aorta tissue derived from Sgk1 knockout mice. LC3 lipidation was analysed by Western blotting (upper panel) and quantified (lower panels) in protein extracts from the heart (a) or aorta (b) of 6 Sgk1 knockout (*Sgk1*−/−) and wild-type (*Sgk1*+/+) mice. Of those, 3 mice were either fed (F) or starved (S) as indicated (sample ID). LC3-positive control (Ctrl) was used to indicate the migration of nonlipidated LC3-I and lipidated LC3-II. Red lines on the box plots (lower panel, left) represent the mean values of all 6 Sgk1 knockout (*Sgk1*−/−) and wild-type (*Sgk1*+/+) mice (lower panel, left) or with regard to fed and starved conditions (lower panel, right). *p* values are provided. Supporting data is provided (Suppl. Data Set).

**Figure 4:**
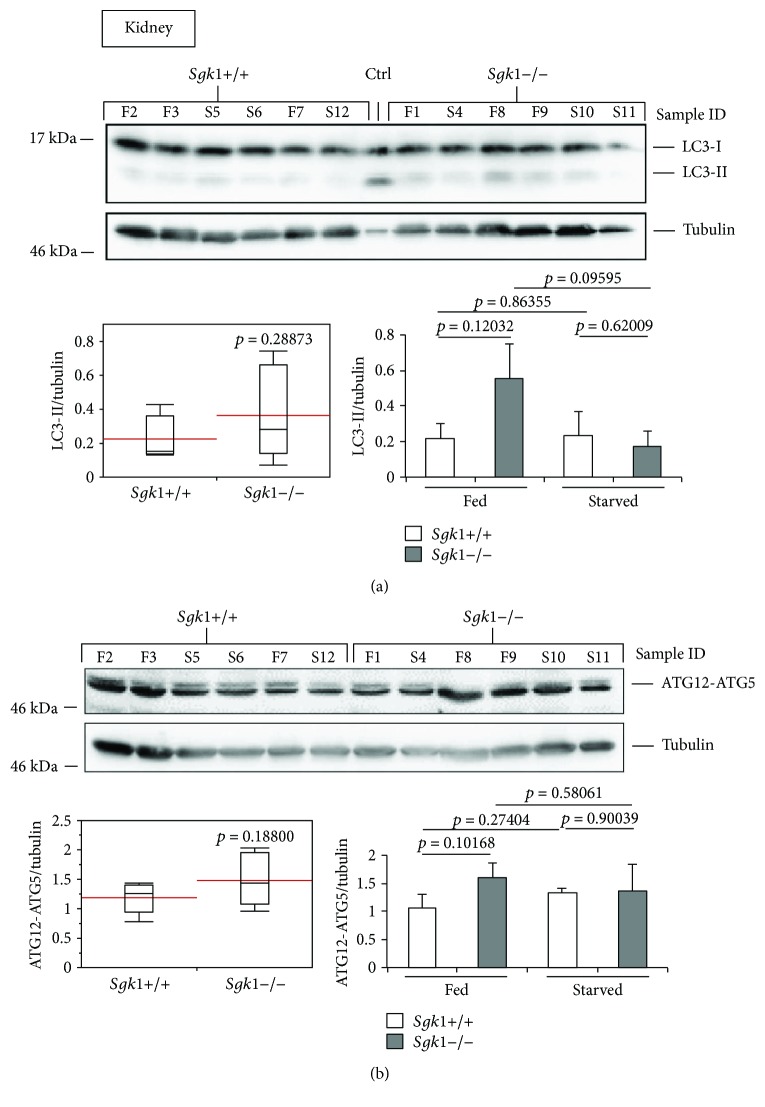
LC3-II and ATG12-ATG5 abundances are unaltered in the mouse kidney of Sgk1 knockout mice. LC3 lipidation (a) and ATG12-ATG5 (b) abundances were analysed by Western blotting (upper panel) and quantified (lower panels) using Sgk1 knockout (*Sgk1*−/−) and wild-type (*Sgk1*+/+) mice (*n* = 6). Of those, mice were either fed (F, *n* = 3) or starved (S, *n* = 3) as indicated (sample ID). LC3-positive control (Ctrl) was used to indicate the migration of nonlipidated LC3-I and lipidated LC3-II. Red lines on the box plots (lower panel, left) represent the mean values of all 6 Sgk1 knockout (*Sgk1*−/−) and wild-type (*Sgk1*+/+) mice (lower panel, left) or with regard to fed and starved conditions (lower panel, right). *p* values are provided. Supporting data is provided (Suppl. Data Set).

To investigate the effect of SGK1 in autophagy, we extracted proteins from liver, kidney, heart, aorta, red and white muscle of SGK1 knockout (*Sgk1*−/−) and wild-type (*Sgk1*+/+) mice (*n* = 6) that were either fed (*n* = 3) or starved (24 h, *n* = 3) prior to cervical dislocation and tissue dissection. Subsequently, we analysed the abundance of the autophagy marker LC3-II (Figures 1 [Fig fig2] [Fig fig3]–4) and ATG12-ATG5 (Figures 1 and 4) by quantitative Western blotting.

We found that the abundances of both LC3-II (Figure 1(a), upper panel and lower left panel) and ATG12-ATG5 (Figure 1(b), upper panel and lower left panel) were significantly increased in red muscle tissue derived from mice deficient for SGK1 (*Sgk1*−/−). In particular, this increase was found when fed mice were only compared with regard to LC3-II abundances (Figure 1(a), lower right panel: *Sgk1*+/+ versus *Sgk1*−/−, fed). Of note, red muscle tissue was the only organ with which we detected a significant difference between fed and starved conditions in the SGK1 wild-type background with regard to LC3-II (Figure 1(a), lower right panel: *Sgk1*+/+, fed versus starved). Based on this, we provide comparisons of SGK1 wild-type and deficient mice irrespective of their nutritional status (Figures 1 [Fig fig2] [Fig fig3]–4, lower left panels) or with regard to fed and starved conditions (Figure 1 [Fig fig2] [Fig fig3]–4, lower right panels). Raw data and statistical analyses for Figures 1 [Fig fig2] [Fig fig3]–4 are provided (Suppl. Data Set).

In the white muscle tissue (Figure 2(a)) and liver (Figure 2(b)), we observed only a weak difference between *Sgk1*+/+ and *Sgk1*−/− mice, apparently when starved conditions were compared in white muscle tissue (Figure 2(a), lower right panel: *Sgk1*+/+ versus *Sgk1*−/−, starved) or fed conditions in liver (Figure 2(b), lower right panel: *Sgk1*+/+ versus *Sgk1*−/−, fed). Furthermore, in the heart (Figure 3(a)), an increase in LC3-II was observed (Figure 3(a), upper panel), but this increase was not significant (Figure 3(a), lower left panel: *p* value 0.06769). In the aorta, an increase of nonconjugated LC3 (LC3-I) was apparent (Figure 3(b), upper panel) but this had no influence on the abundance of LC3-II, which was not significant when *Sgk1*+/+ and *Sgk1*−/− mice were compared (Figure 3(b), lower panels). In the kidney tissue (Figure 4), we observed a slight increase in LC3-II; however, this was found not to be significant (Figure 4(a), lower panels), consistent with no significant alterations with regard to ATG12-ATG5 (Figure 4(b)).

In summary, our tissue screening approach assigned an inhibitory role of SGK1 on autophagy in the red muscle tissue.

### 3.2. SGK1 Inhibits Autophagy In Vitro

**Figure 5:**
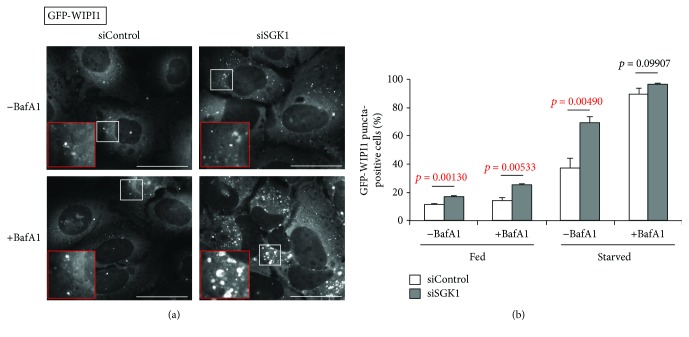
SGK1 silencing provokes an increase of GFP-WIPI1 puncta. Stable GFP-WIPI1 expressing U-2 OS cells were transiently transfected with control siRNA (siControl) or siSGK1 and fed or starved for 3 h with or without bafilomycin A1 (BafA1). (a) Representative images for starved conditions with and without BafA1 treatment are shown. Scale bars 40 *μ*m. (b) The percentage GFP-WIPI puncta cells were calculated (up to 1541 cells per condition, *n* = 3). *p* values are provided. Supporting data is provided (Suppl. Data Set).

**Figure 6 fig6:**
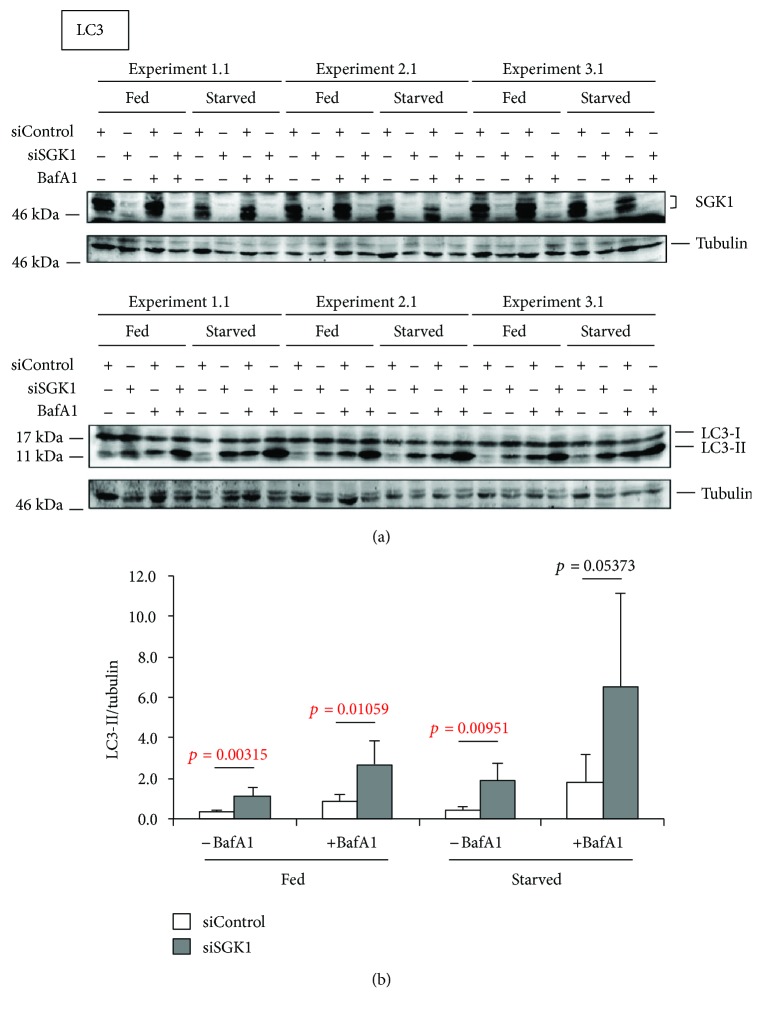
Elevated LC3 lipidation in SGK1-depleted cells. Stable GFP-WIPI1 expressing U-2 OS cells were transiently transfected with control siRNA (siControl) or siSGK1 and fed or starved for 3 h with or without bafilomycin A1 (BafA1). (a) SGK1 depletion was confirmed by Western blotting (*n* = 6, 3 independent experiments are shown). The autophagic flux was assessed by LC3 (LC3-II) Western blot analysis. (b) LC3-II levels were normalized over tubulin (*n* = 6, 3 experiments are shown). *p* values are provided. Supporting data is provided (Suppl. Data Set).

**Figure 7:**
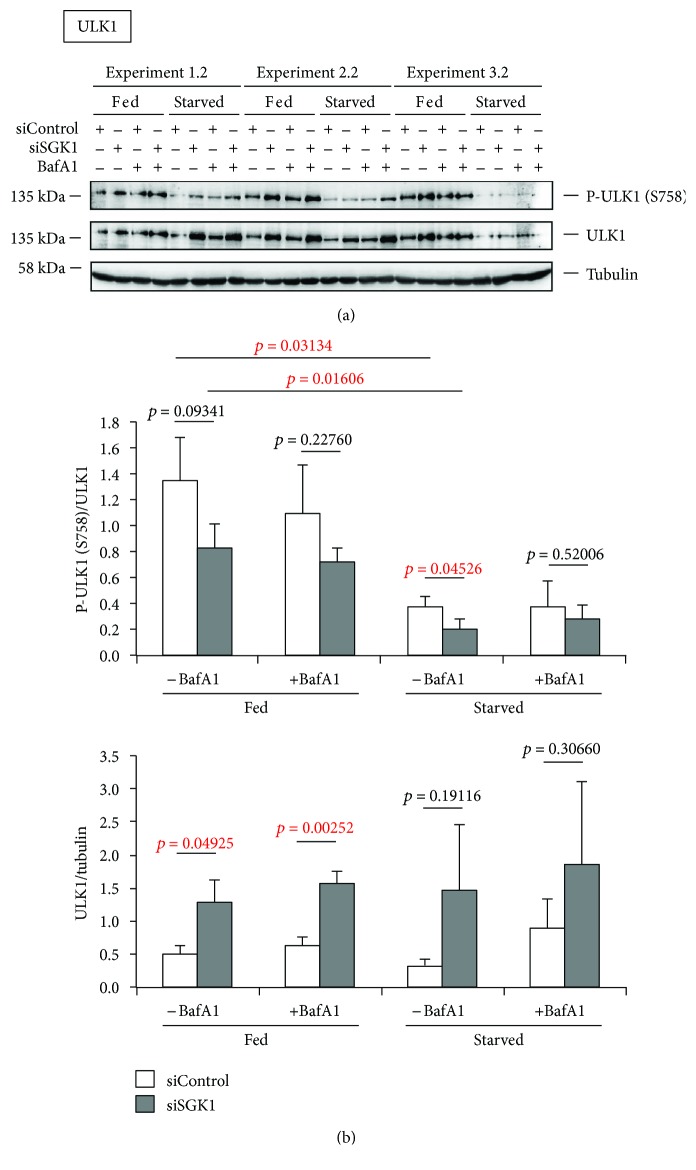
Increased ULK1 protein abundance in SGK1-depleted cells. Stable GFP-WIPI1 expressing U-2 OS cells were transiently transfected with control siRNA (siControl) or siSGK1 and fed or starved for 3 h with or without bafilomycin A1 (BafA1). (a) Phosphorylated and total ULK1 protein was detected by sequential Western blotting. (b) Phospho-ULK1 (S758)/ULK1 and ULK1/tubulin ratios were quantified (*n* = 3, in duplicates). *p* values are provided. Supporting data is provided (Suppl. Data Set).

To investigate the role of SGK1 *in vitro*, we used a U-2 OS cell model established for the specific detection of autophagosomal membranes (puncta) decorated with the WIPI1 PI3P effector in autophagy [9]. Of note, due to its low endomembrane content, the U-2 OS cell line is superior for the fluorescence-based detection of newly formed membranes, such as autophagosomes. Here, we transiently transfected U-2 OS cells stably expressing GFP-WIPI1 with siRNAs targeting endogenous SGK1 (siSGK1) [29], along with nontargeting control siRNAs (siControl) (Figures 5 [Fig fig6]–7). To adhere to the standard conditions that modulate autophagy, we used fed conditions (Fed), as well as starvation conditions (Starved) where we treated the cells with EBSS for maximal induction of autophagy *in vitro* [8], and to distinguish between an induction or a block of autophagy, we applied the lysosomal inhibitor bafilomycin A1 [10, 30] (Figures 5 [Fig fig6]–7).

As expected, starvation conditions elevated the number of cells displaying autophagosomal membranes with GFP-WIPI1 that further increased when lysosomes were inhibited (+BafA1) (Figure 5(a), 5(b)). Further, we observed that upon SGK1 silencing (siSGK1) GFP-WIPI1 puncta prominently increased (Figure 5(a); representative images from starved cells are shown). This observation became even more apparent when we calculated (up to 1541 cells per treatment, *n* = 3) the number of GFP-WIPI1 puncta-positive cells treated either with siControl or with siSGK1 (Figure 5(b)). In both fed and starved conditions, the absence of SGK1 induced a significant increase of GFP-WIPI1-harboring autophagosomes (puncta) (Figure 5(b)). Importantly, we confirmed prominent silencing of endogenous SGK1 in all applied conditions by parallel Western blotting (*n* = 6, 3 experiments are shown in Figure 6(a)). Subsequently, we assessed the abundance of lipidated, autophagosomal LC3 (LC3-II) in both the presence (siControl) or the absence of endogenous SGK1 (siSGK1) in fed and starved conditions with or without lysosomal inhibitor (BafA1) (*n* = 6, 3 independent experiments are shown in Figure 6(a)). Again, we found that in cells with downregulated SGK1 (siSGK1), more LC3-II protein was present, in fed and also in starved conditions (Figure 6(a), lower panels; Figure 6(b)). Importantly, in conditions where we employed bafilomycin A1 (BafA1), the abundances of LC3-II further increased, demonstrating that not only more lipidated LC3 (LC3-II) was produced in the absence of SGK1, but also more autophagic flux was observed (Figure 6(b)). In addition, we assessed the abundance of ULK1 protein upon SGK1 silencing and also in parallel its phosphorylation status with regard to the inhibitory phosphorylation by TORC1 (S758) (Figure 7(a); *n* = 3 with duplicates; one of each duplicate is presented). As expected, we found that upon starvation, less ULK1 was phosphorylated at S758 (Figure 7(b), upper panel). Interestingly, we further found that ULK1 protein levels increased in the absence of SGK1 (Figures 7(a) and 7(b) lower panel), but not its phosphorylation at serine 758 (Figure 7(b)). In fact, upon starvation, significantly less ULK1 was phosphorylated in the absence of SGK1 (Figure 7(b)).

## 4. Discussion

To investigate the role of SGK1 in autophagy, we analysed the abundance of the autophagy markers LC3-II and ATG12-ATG5 in organ tissues derived from SGK1 knockout and wild-type mice. We also downregulated endogenous SGK1 in U-2 OS GFP-WIPI1 cells and analysed autophagic activity *in vitro*.

Our *in vivo* analysis using quantitative LC3-II and ATG12-ATG5 Western blotting suggests that in SGK1 knockout mice autophagic activity is significantly increased in red muscle tissue (Figure 1) and weakly in the white muscle and the liver (Figure 2). As increased levels of LC3-II and ATG12-ATG5 suggest increased autophagic activity, SGK1 likely inhibits autophagy in these tissues.

Our *in vitro* analysis using quantitative LC3-II Western blotting and GFP-WIPI1 puncta analysis confirmed that SGK1 should inhibit the process of autophagy. We observed a significant increase of GFP-WIPI1-bound autophagosomes (puncta). This demonstrates that the absence of SGK1 provokes the induction of autophagy via the canonical requirement for newly produced PI3P, which is then bound by WIPI1/2 proteins (here GFP-WIPI1) to mediate the recruitment of the ATG12-ATG5/ATG16L complex for subsequent LC3 lipidation (production of LC3-II) [5].

Indeed, when we assessed LC3 lipidation in cells depleted from endogenous SGK1, a significant increase of LC3-II was confirmed. This was found in both fed and starved conditions, showing that SGK1 should inhibit basal as well as induced autophagy. We further addressed the question if the absence of SGK1 would additionally provoke an increase of the autophagic flux. These assessments were carried out using fed and starved conditions in both the presence or the absence of bafilomycin A1, which inhibits lysosomal function hence blocking autolysosomal degradation. Indeed, in the absence of SGK1, LC3-II levels (and also GFP-WIPI1 puncta) significantly increased, suggesting that the autophagic flux was also stimulated without SGK1. Taken together, both our *in vivo* and *in vitro* analyses of autophagy support the notion that SGK1 should function as a negative regulator of autophagosome formation and autophagic degradation.

Additionally, we also asked whether or not we could observe a difference in ULK1 protein level and its phosphorylation status in the absence of SGK1. Interestingly, we observed that upon SGK1 depletion more ULK1 protein was detected, indicating that SGK1 should contribute to the regulation of ULK1 gene expression, a topic of recent interest [31]. Moreover, as more ULK1 protein was present in the absence of SGK1, not more (but even less) phosphorylation of ULK1 at serine 758 occurred. TORC1 targets serine 758 in ULK1 for phosphorylation, resulting in the inhibition of ULK1. A decrease in ULK1 phosphorylation at serine 758 hence liberates ULK1 protein to initiate autophagy, which in fact was observed in the absence of SGK1 (see above). In general, ULK1 mRNA and protein levels are fine-tuned during the process of autophagy, showing that ULK1 availability is tightly controlled at different, as yet unidentified, stages [32].

**Figure 8:**
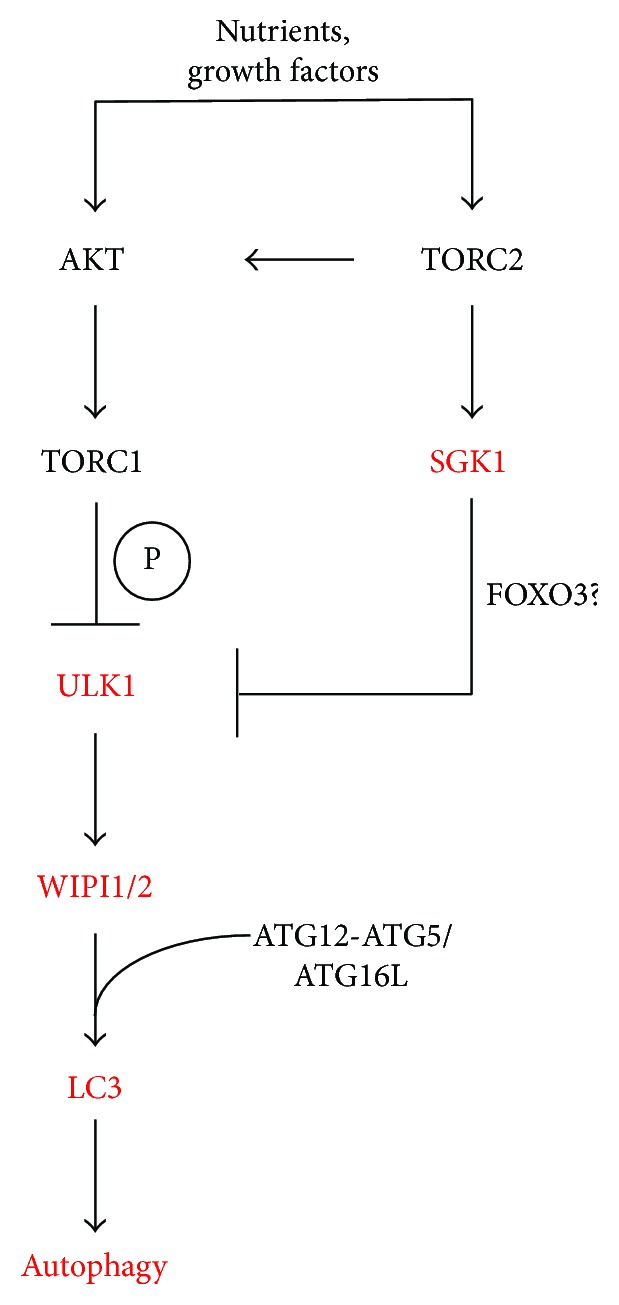
Working model for the role of SGK1 in the control of autophagy.

We hypothesize (Figure 8) that SGK1 may regulate autophagy by contributing to regulate ULK1 protein level, perhaps through modulating ULK1 gene expression. Interestingly, the transcription factor FOXO3 was found to be specifically phosphorylated by SGK1, which results in the nuclear exclusion of FOXO3 hence its inhibition to function as a transcription factor [27, 33]. FOXO3, however, is required to initiate ULK1 gene expression as previously shown [34]. Based on this, it is tempting to speculate that SGK1 may regulate ULK1 gene expression via FOXO3 (Figure 8).

## Supplementary Materials

Supplementary Materials Supporting data is provided in the Supplementary Data Set (Suppl. Data Set). As indicated in each figure legend, the Suppl. Data Set (excel file) includes statistical analyses with *p* values for the following main figures: Figures 1(a), 1(b), 2(a), 2(b), 3(a), 3(b), 4(a), 4(b), 5(b), 6(b), and 7(b). Individual excel sheet labels correspond to the main figure labels.
